# Precision medicine in the treatment of colorectal cancer with liver metastases

**DOI:** 10.20892/j.issn.2095-3941.2023.0483

**Published:** 2024-02-05

**Authors:** Yuqiu Xu, Yang Lv, Zhehui Zhu, Yijiao Chen, Peiwen Zhou, Lechi Ye, Wentao Tang, Jianmin Xu

**Affiliations:** Department of Colorectal Surgery, Zhongshan Hospital, Fudan University, Shanghai 200032, China

Colorectal cancer (CRC), one of the most common malignant diseases, ranks second in morbidity and third in mortality among all cancers worldwide^1^. The liver is the most common site of distant metastasis in CRC. Liver metastasis is also the main cause of death in patients with CRC. Approximately 25% of patients with CRC have liver metastasis detected at the initial diagnosis, and approximately 50% of patients eventually develop liver metastasis during disease progression. Liver metastasis severely affects the prognosis of patients with CRC. In recent years, with deepening understanding of the occurrence and development of CRC liver metastasis (CRLM), many targeted drugs have been approved for use, and surgical techniques have increasingly been optimized. Although the prognosis of patients has improved, overall treatment efficacy remains unsatisfactory^2^.

CRLM is a heterogeneous disease whose clinical and pathological characteristics, and treatment responses, vary among patients. Tumor biological behavior also plays a crucial role in influencing efficacy and prognosis. Therefore, targeted implementation of individualized treatments is critical in the era of precision medicine, thus necessitating precise diagnosis, classification, and treatment at both the clinical and molecular levels. Herein, we summarize the current status of precision treatment for CRLM and the future outlook regarding developments and trends.

## Multidisciplinary treatment (MDT)

MDT refers to the development of personalized treatment plans for patients through joint discussions among senior experts from multiple related disciplines. MDT is recommended for patients with CRLM. Before treatment, patients undergo comprehensive evaluation by a team composed of experts in surgery; pathology; oncology; radiology; radiation therapy; and related disciplines, such as thoracic surgery, urology, and gynecology. Patient performance status, age, underlying diseases, tumor molecular pathological characteristics, and other conditions are evaluated in a personalized manner. MDT has been demonstrated to increase the accuracy of diagnosis, efficacy of adherence and standardized treatment, and rate of liver conversion resection. The overall survival benefits of MDT are clearer in patients with higher clinical risk scores^3^. Therefore, MDT is the best treatment mode for CRLM precision therapy.

## Local treatment

Surgical removal of liver metastases is currently the best method to cure CRLM. To avoid postoperative liver failure, a sufficient future liver remnant (FLR) must be retained during surgery. Generally, for patients with normal liver function, the FLR must account for more than 25% of the total liver volume. For patients with liver cirrhosis, the FLR must account for more than 40% of the total liver volume^4^. Because of an inadequate FLR, less than 25% of patients are eligible for surgery at diagnosis. Many new technologies have been developed to optimize the FLR. Portal vein embolization was the first such technology to be introduced. However, the effect of liver hypertrophy is poor, and the waitng time for sufficient FLR is too long; consequently, patients may not be able to undergo two-stage surgery. Associated liver partition and portal vein ligation for staged hepatectomy (ALPPS) was later introduced. A rapid increase in FLR caused by ALPPS improves the tumor resection rate. However, more complication and mortality have also been reported with this method. Therefore, the application of ALPPS has long been controversial. Liver venous deprivation, a new procedure that has received widespread attention, achieves faster FLR increases than PVD, and lower complication and mortality than ALPPS. However, this new technology requires additional clinical experience and further standardization of operation methods.

Radiofrequency ablation (RFA), a local ablation therapy, has become increasingly important in the field of CRLM, because of its advantages of minimal invasiveness and effects on liver function, and fast recovery.

RFA treatment for small diameter initial resectable CRLM has been shown to have equivalent efficacy to that of surgical resection^5^. However, other studies have shown that the intrahepatic recurrence rate after RFA treatment is higher than that after surgery^6^. Controversy persists surrounding initially resectable CRLM treated with RFA, and relevant randomized controlled trials are urgently needed to provide confirmation. For unresectable CRLM, surgery combined with RFA provides some patients with an opportunity for curative treatment, and achieves better prognosis than palliative chemotherapy. RFA can also induce exposure to tumor antigens and generate tumor specific T cell responses, thereby enhancing the body’s anti-tumor immune function. The combination of RFA and tumor immunotherapy can have synergistic effects, thus leading to new ideas for the treatment of CRLM.

Beyond RFA, other thermal ablation techniques include microwave ablation, laser hyperthermia, high-intensity focused ultrasound ablation, and cryoablation. The principles rely on various technical means to transmit energy to tumor sites and cause local temperatures to rise or fall, thereby killing tissue cells. Nonthermal ablation techniques use other principles to achieve cell damage, such as chemical ablation (such as alcohol ablation) or stereotactic body radiation therapy—an external irradiation technique with high precision and conformability, which can achieve therapeutic effects similar to those of surgical resection.

## Immunotherapy

The presence of microsatellite instability high (MSI-H) or mismatch-repair deficiency (dMMR) has been identified in approximately 15% of CRC cases. These frequencies are higher in localized CRC than metastatic CRC (mCRC), (approximately 5% in stage IV). MSI-H CRC has a high mutational burden, thus producing large amounts of new antigens, which in turn induce anti-tumor immune responses. The body resists this response by upregulating immunosuppressive signals (such as PD-1/PD-L1), thus preventing tumor clearance. Immune checkpoint inhibitors disinhibit T cell function and consequently lead to tumor killing.

In KEYNOTE-177, a milestone study of immunotherapy for mCRC, treatment with pembrolizumab (inducing PD-1 blockade) was associated with significantly longer progression-free survival than chemotherapy (median, 16.5 *vs*. 8.2 months; hazard ratio, 0.60; *P* = 0.0002) in patients with mCRC with MSI-H/dMMR who had not previously received treatment. The overall response was 43.8% in the pembrolizumab group and 33.1% in the chemotherapy group^7^. The KEYNOTE-177 study was the first randomized study to demonstrate the clinical benefits of PD-1 blockers as a first-line treatment in patients with mCRC with MSI-H/dMMR. The findings support pembrolizumab as a first-line treatment for patients with mCRC with MSI-H/dMMR.

Beyond pembrolizumab, other antibodies to PD-1, such as nivolumab, have shown efficacy in treating patients with mCRC with MSI-H/dMMR. In the CheckMate 142 series study, a combination of nivolumab with ipilimumab, another immune checkpoint inhibitor, showed clinical benefits in patients with MSI-H/dMMR as a first-line or later-line treatment.

Because immunotherapy has become the standard treatment in patients with mCRC with MSI-H/dMMR, MMR/MSI testing is required in every patients with CRC.

Approximately 95% of mCRC is classified as mismatch repair proficient (pMMR)/microsatellite stable (MSS), and the response to immunotherapy is poor. Improving the efficacy of immunotherapy in these patients is a major focus. Currently, multiple combination therapy modalities are used in clinical practice for mCRC with pMMR/MSS. Studies have combined an immune checkpoint inhibitor with a tyrosine kinase inhibitor, anti-VEGF antibodies, anti EGFR antibodies, or inhibitors of mitogen-activated protein kinase (MAPK) signaling. The REGONIVO study explored a combination of immunotherapy and targeted therapy for pMMR/MSS mCRC, but the results have not been successfully replicated in other clinical trials. Additionally, the benefit for patients with liver metastases is poor. A combination of 2 immune checkpoint inhibitors is expected to improve efficacy, but most current research is in early stages.

In summary, for patients with MSI-H/dMMR, immunotherapy is recommended as a first-line, second-line, or later-line treatment. However, for patients with pMMR/MSS, particularly those with CRLM, immunotherapy remains a major challenge.

## Targeted therapy

To achieve precision medicine-based treatment for each patient, many studies have sought to identify druggable mutations. Targeting the epidermal growth factor receptor (EGFR) family and its intracellular signaling pathways, including the RAS-RAF-MEK-MAPK pathway, has become an essential component of molecular targeted therapies for CRLM.

The RAS family includes HRAS, NRAS, and KRAS. More than 40% of mCRC cases have KRAS mutations. In RAS mutated cases, doublet (FOLFOX/FOLFIRI) or triplet (FOLFOXIRI) chemotherapy with bevacizumab, an antibody to vascular endothelial growth factor (VEGF), is recommended^8^. In the treatment of RAS wild-type cases, primary tumor location is an important prognostic biomarker. The benefit of anti-EGFR in combination with chemotherapy is limited to left-sided RAS wild-type cases. Recently, inhibitors of KRAS, which was considered undruggable, have achieved a breakthrough. Two drugs, sotorasib and adagrasib, have been approved by the FDA for the treatment of G12C-mutant KRAS; this mutation occurs in approximately 3%–4% of patients with mCRC. Although monotherapy for KRAS G12C inhibition has shown only modest anti-tumor activity in the treatment of mCRC, a KRAS G12C inhibitor plus an EGFR inhibitor has been found to significantly improve the response. In The CodeBreaK 300 trial, the median progression-free survival was 5.6 months, 3.9 months, and 2.2 months in the 960-mg sotorasib-panitumumab, 240-mg sotorasib-panitumumab, and trifluridine-tipiracil or regorafenib groups, respectively; moreover, the objective response was 26.4%, 5.7%, and 0%, respectively^9^. In addition, the anti-tumor efficacy of MRTX1133, a KRAS G12D inhibitor, is being evaluated in the treatment of KRAS G12D-mutated CRC.

BRAF mutations, predominantly V600E alterations, occur in approximately 10% of mCRC cases. BRAF V600E mutated mCRC is associated with poor prognosis and an unsatisfactory response to therapy. Doublet or triplet chemotherapy, with the addition of bevacizumab is often recommended. In addition to traditional chemotherapy regimens, BRAF inhibitors (including vemurafenib, dabrafenib, and encorafenib) and MEK inhibitors (including trametinib, cobimetinib, and binimetinib) have shown promising activity in BRAF V600E mutated tumors. However, monotherapy with these inhibitors has not been successful in mCRC, possibly because of the negative feedback on EGFR and paradoxical activation of the MAPK pathway. The BEACON trial, in patients with *BRAF* V600E-mutated mCRC who experienced disease progression after 1 or 2 previous regimens, has reported that both the doublet-therapy (encorafenib and cetuximab) and triplet-therapy (encorafenib, binimetinib, and cetuximab) groups had significantly longer median overall survival than the control group (receiving either cetuximab and irinotecan or cetuximab and FOLFIRI)^10^. The SWOG S1406 trial has also indicated that vemurafenib in combination with cetuximab and irinotecan (VIC regimen) is effective in BRAF V600E-mutated mCRC previously treated with 1 or 2 regimens^11^. BRAF mutation is also characterized by MSI-H, and 21%–46% of BRAF-mutant CRC cases are associated with dMMR. Some patients may benefit from treatment with immune checkpoint inhibitors. The SEAMARK trial, a phase II study, is exploring the benefit of encorafenib and cetuximab plus anti-PD-1 pembrolizumab as a first-line therapy for MSI-H and BRAF V600E-mutant mCRC^12^.

HER2 gene amplification is relatively rare in mCRC (approximately 3% of cases) and is commonly observed in RAS/BRAF wild-type tumors. HER2 overexpression usually causes resistance to anti-EGFR therapies. Targeting HER2 has been found to improve prognosis in HER2-positive breast cancer. The MyPathway trial has assessed the activity of pertuzumab and trastuzumab in patients with HER2-amplified mCRC, and confirmed the utility of dual HER2 targeting therapy, with an ORR of 32%^13^. Anti-HER2 trastuzumab plus tyrosine kinase inhibitor treatment has also shown promising clinical application prospects. The MOUNTAINEER study treated chemotherapy-refractory, HER2-positive, RAS WT unresectable, or mCRC with tucatinib plus trastuzumab, and found an ORR per BICR of 38.1%^14^. Additionally, antibody-drug conjugate is a potential therapy for HER2-positive mCRC. The DESTINY-CRC01 trial, using trastuzumab-deruxtecan (DS-8201), has confirmed an ORR of 45.3%, and promising and durable activity^15^.

## Discussion and perspectives

In this Editorial, we summarized the development of precision treatment for CRLM. Some patients can be cured by resection of liver metastases. However, the type of liver resection (anatomical resection or non-anatomical resection) for CRLM remains under debate. Patients with CRLM with gene mutation or right-sidedness can benefit more from anatomical resection than non-anatomical resection^16^. More prospective studies are needed. Tumor biological behavior should be further explored. Most liver metastases are unresectable, and several new local treatments have been proposed. Radioembolization using ^90^Y microspheres is one such treatment. Two types of ^90^Y microspheres are available: glass microspheres, which might be more suitable for hepatocellular carcinoma with portal vein invasion and radiation segmentectomy, and resin microspheres, which might be more suitable for larger tumors and those with high arterial flow^17^. Proton beam therapy (PBT) is another option that has been demonstrated to be effective and safe for CRLM. In a phase II trial of hypofractionated high-dose PBT for unresectable LM, in which the colorectum accounted for 60% of the primary cancer sites, the rate of 6-month freedom from local progression was 95.2%^18^. Some problems remain, such as dosage and safety issues, and additional randomized controlled trials are needed to improve both ^90^Y microsphere radioembolization and PBT therapies. Liver transplantation is another research focus. In the SECA-II study, patients with nonresectable liver-only CRC receiving liver transplantation achieved an overall survival of 100%, 83% and 83% at 1, 3, and 5 years, respectively^19^. To prolong survival with liver transplantation, a consensus must be reached regarding several key domains, such as patient selection, evaluation of biological behavior, and organ selection and allocation.

Current precision treatments with immunotherapy and targeted therapies for CRLM are based on limited molecular (MSI/RAS/RAF/HER2) markers. However, owing to the complex and heterogeneous of CRLM and the undruggability of most oncogenic divers, the current treatment remains unsatisfactory for most patients. For example, in the treatment of patients with dMMR mCRC with anti-PD-1, liver metastasis is associated with poorer PFS than non-liver metastasis. Thus, future studies will need to re-explore multi-drug combination regimens of existing molecular markers and find more druggable targets. Trials are increasingly being conducted to assess the efficacy and safety of immunotherapy in combination with chemotherapy or targeted therapies. Cancer vaccines, adoptive cell therapy, and gut microbiota regulation are potential means of improving host and tumor microenvironments, and the responses of primary tumor and liver metastases to treatment regimens. New drug delivery methods, such as autophagy receptor mediated nanodrug delivery, have been found to enhance the efficacy of albumin-bound paclitaxel for CRC. Single-cell sequencing technology aids in integrating tumor gene alteration information; analyzing the composition of the immune microenvironment; and exploring new molecular markers, immune cell subtypes, and immune checkpoints^20^.

## References

[1] Siegel RL, Miller KD, Wagle NS, Jemal A (2023). Cancer statistics, 2023. CA Cancer J Clin.

[2] Jiang Y, Yuan H, Li Z, Ji X, Shen Q, Tuo J (2021). Global pattern and trends of colorectal cancer survival: a systematic review of population-based registration data. Cancer Biol Med.

[3] Lv Y, Feng QY, Wei Y, Ren L, Ye Q, Wang X (2020). Benefits of multi-disciplinary treatment strategy on survival of patients with colorectal cancer liver metastasis. Clin Transl Med.

[4] Kishi Y, Vauthey JN (2021). Issues to be considered to address the future liver remnant prior to major hepatectomy. Surg Today.

[5] Kron P, Linecker M, Jones RP, Toogood GJ, Clavien PA, Lodge JPA (2019). Ablation or resection for colorectal liver metastases? A systematic review of the literature. Front Oncol.

[6] Schullian P, Johnston EW, Putzer D, Laimer G, Waroschitz G, Braunwarth E (2021). Stereotactic radiofrequency ablation (SRFA) for recurrent colorectal liver metastases after hepatic resection. Eur J Surg Oncol.

[7] André T, Shiu KK, Kim TW, Jensen BV, Jensen LH, Punt C (2020). Pembrolizumab in microsatellite-instability-high advanced colorectal cancer. N Engl J Med.

[8] Cervantes A, Adam R, Roselló S, Arnold D, Normanno N, Taïeb J (2023). Metastatic colorectal cancer: ESMO clinical practice guideline for diagnosis, treatment and follow-up. Ann Oncol.

[9] Fakih MG, Salvatore L, Esaki T, Modest DP, Lopez-Bravo DP, Taieb J (2023). Sotorasib plus panitumumab in refractory colorectal cancer with mutated KRAS G12C. N Engl J Med.

[10] Kopetz S, Grothey A, Yaeger R, Van Cutsem E, Desai J, Yoshino T (2019). Encorafenib, binimetinib, and cetuximab in BRAF V600E-mutated colorectal cancer. N Engl J Med.

[11] Kopetz S, Guthrie KA, Morris VK, Lenz HJ, Magliocco AM, Maru D (2021). Randomized trial of irinotecan and cetuximab with or without vemurafenib in BRAF-mutant metastatic colorectal cancer (SWOG S1406). J Clin Oncol.

[12] Elez E, Kopetz S, Tabernero J, Bekaii-Saab T, Taieb J, Yoshino T (2023). SEAMARK: phase II study of first-line encorafenib and cetuximab plus pembrolizumab for MSI-H/dMMR BRAFV600E-mutant mCRC. Future Oncol (London, England).

[13] Meric-Bernstam F, Hurwitz H, Raghav KPS, McWilliams RR, Fakih M, VanderWalde A (2019). Pertuzumab plus trastuzumab for HER2-amplified metastatic colorectal cancer (MyPathway): an updated report from a multicentre, open-label, phase 2a, multiple basket study. Lancet Oncol.

[14] Strickler JH, Cercek A, Siena S, André T, Ng K, Van Cutsem E (2023). Tucatinib plus trastuzumab for chemotherapy-refractory, HER2-positive, RAS wild-type unresectable or metastatic colorectal cancer (MOUNTAINEER): a multicentre, open-label, phase 2 study. Lancet Oncol.

[15] Siena S, Di Bartolomeo M, Raghav K, Masuishi T, Loupakis F, Kawakami H (2021). Trastuzumab deruxtecan (DS-8201) in patients with HER2-expressing metastatic colorectal cancer (DESTINY-CRC01): a multicentre, open-label, phase 2 trial. Lancet Oncol.

[16] Chang W, Chen Y, Zhou S, Ren L, Xu Y, Zhu D (2023). Anatomical resection improves relapse-free survival in colorectal liver metastases in patients with KRAS/NRAS/BRAF mutations or right-sided colon cancer: a retrospective cohort study. Int J Surg (London, England).

[17] Boas FE, Bodei L, Sofocleous CT (2017). Radioembolization of colorectal liver metastases: indications, technique, and outcomes. J Nucl Med.

[18] Kim K, Yu JI, Park HC, Yoo GS, Lim DH, Noh JM (2022). A phase II trial of hypofractionated high-dose proton beam therapy for unresectable liver metastases. Radiother Oncol.

[19] Dueland S, Syversveen T, Solheim JM, Solberg S, Grut H, Bjørnbeth BA (2020). Survival following liver transplantation for patients with nonresectable liver-only colorectal metastases. Ann Surg.

[20] Xu Y, Wei Z, Feng M, Zhu D, Mei S, Wu Z (2022). Tumor-infiltrated activated B cells suppress liver metastasis of colorectal cancers. Cell Rep.

